# Reproductive biology of the pampas deer (*Ozotoceros bezoarticus*): a review

**DOI:** 10.1186/1751-0147-50-16

**Published:** 2008-06-05

**Authors:** Rodolfo Ungerfeld, Solana González-Pensado, Alejandro Bielli, Matías Villagrán, Daniel Olazabal, William Pérez

**Affiliations:** 1 Departamento de Fisiología, Facultad de Veterinaria, Montevideo, Uruguay; 2 Departamento de Morfología y Desarrollo, Facultad de Veterinaria, Montevideo, Uruguay

## Abstract

The pampas deer (*Ozotoceros bezoarticus*) is a South American grazing deer which is in extreme danger of extinction. Very little is known about the biology of the pampas deer. Moreover, most information has not been published in peer-reviewed scientific journals, and is only available in local publications, theses, etc. Therefore, our aim was to update and summarize the available information regarding the reproductive biology of the pampas deer. Moreover, in most sections, we have also included new, unpublished information. Detailed descriptions are provided of the anatomy of both the female and the male reproductive tract, puberty onset, the oestrous cycle and gestational length. Birthing and the early postpartum period are described, as are maternal behaviour and early fawn development, seasonal distribution of births, seasonal changes in male reproduction and antler cycle, reproductive behaviour, semen collection, and cryopreservation. Finally, an overview is given and future directions of research are proposed.

## Background

The pampas deer, *Ozotoceros bezoarticus *(Linnaeus, 1758), used to be a widespread species originally distributed in the open grasslands (pampas and savannas) in eastern South America, from 5° to 41° S [1]. In the 1800s naturalists and voyagers reported great abundance of this species [1-3]. It was the most widespread cervid in Uruguay [3]. Reports of explorers and pioneer settlers as well as the folklore clearly tell how the pampas deer could be found in larger groups throughout the grasslands during the 17^th ^and 18^th ^centuries. Even place names in the region bear witness to the widespread distribution of this species. Some records report that more than 2,300,000 deer skins were exported during the 19^th ^century from the Río de la Plata [4]. However, due to man's direct and indirect influence the population has decreased substantially in both size and distribution. This decrement has been explained by habitat fragmentation, agricultural development and competition with farmed animals [5], unregulated hunting [6] and transmission of infectious diseases [7].

Over the past few decades, the isolation of the few remaining populations has pushed this species to the brink of extinction. Small populations have been reported in Argentina (Bahía Samborombón [8], Corrientes [9], San Luis [10], Santa Fé [11]), Brazil [12,13] and Uruguay [14,15]. Although small populations were reported some years ago in Bolivia [16], no up-to-date data about them have been reported. Overall, the pampas deer is considered in extreme danger of extinction. It has been listed in Appendix I of the Convention on International Trade in Endangered Species of Wild Fauna and Flora (CITES) [17] since 1975, and is considered by the International Union for Conservation of Nature and Natural Resources (UICN) as being in critical danger of extinction [18]. The biggest known semi-captive population, made up of approximately 80 individuals (2008), has been bred for the last 25 years at the Estación de Cría de Fauna Autóctona (ECFA), Pan de Azúcar, Uruguay (33°47' S, 54°00' W) [19].

In spite of the fact that this is an endangered species, very little is known about the biology of the pampas deer. Moreover, most information has not been published in peer-reviewed scientific journals, and is only available in local publications, theses, etc. Therefore, our aim was to update and summarize the available information regarding the reproductive biology of the pampas deer, including female and male anatomy, puberty and seasonal reproductive patterns, maternal and sexual-related behaviours and reproductive techniques. New, unpublished information on all these areas will also be included.

## General description of the species

Until recently, only three subspecies of the pampas deer were recognized: *O. bezoarticus bezoarticus*, occurring in Brazil, *O. bezoarticus celer*, in Argentina, and *O. bezoarticus leucogaster*, in southwestern Brazil, northeastern Argentina (Corrientes) and southeastern Bolivia [2]. Cabrera [2] and Jackson [20] were unable to describe the taxonomic characteristics of the Uruguayan populations. The existence of two different subspecies endemic in Uruguay, *O. bezoarticus arerunguaensis *(Salto, northwestern Uruguay) and *O. bezoarticus uruguayensis *(Sierra de Ajos, Rocha, southeastern Uruguay), was described based on cytogenetics and molecular [21] and morphometric [22] data.

Pampas deer males are somewhat bigger than females [20]. Free-ranging males reach a length of 130 cm (muzzle tip to tail base), measuring 75 cm at shoulder height and having a tail length of 15 cm. They weigh approximately 35 kg. However, data obtained from animals bred in semi-captivity indicate slightly smaller animals, with males measuring approximately 90–100 cm long, shoulder height 65–70 cm, and weighing 30–35 kg. Antlers are middle-sized when compared with other deer, solid and thin. Antlers reach 30 cm long, have three points, a brow point and a rear, and a longer bifurcated branch [23]. Females reach 85 cm length and 65 cm at shoulder height, with their body weight being 20–25 kg (unpublished data). Males are usually darker coated than females [2].

## Female reproduction

### Anatomy of the female reproductive tract

The main characteristics of the pampas deer reproductive tract have not been previously described. In this section we present the main anatomical characteristics of tracts obtained from six dead females (*O. bezoarticus arerunguaensis*). Three of them were prepubertal; the other three were adults that died during the spring at the ECFA, Uruguay. Anatomical terms are used according to the Nomina Anatomica Veterinaria [24].

**Figure 1:**
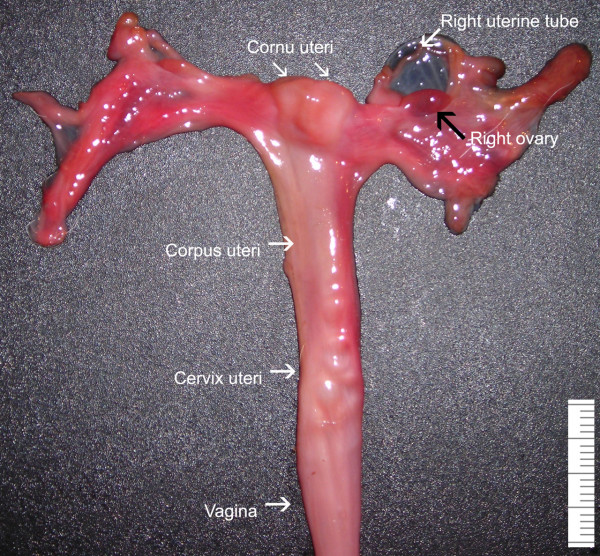
Overview of the female reproductive tract of the pampas deer (*Ozotoceros bezoarticus*).

The female genital organs of *O. bezoarticus *are located inside the pelvic cavity. These organs are small in relation to body size when compared with other cervids, such as the Pere David's deer [25]. Overall, considering the vagina, cervix, corpus and cornua uteri, the tract is elongated (Fig. 1). The same parts as described for domestic ruminants are recognized [24]. Ovaries measure approximately 1.0 × 0.6 × 0.5 cm, and each ovary weighs 0.5 g, with no differences between the right and left ovary. In the ovaries studied no corpus luteum was visible.

The uterine tubes are fairly flexuous, and run together within the mesosalpinx at about 0.5 cm from its free border. The ovarian fimbriae, infundibulum, ampulla and isthmus are clearly differentiated. Between the ampulla and the isthmus the tract narrows in diameter. Colour intensity decreases markedly along the uterine tubes, the distal end being much paler.

The uterus comprises the cervix, corpus and cornua. The uterus is bicornuated, with tapering proximal ends of the cornua. Unlike those in domestic ruminants, the cornua uteri are not spiralled, and are directed laterally, with a slight ventral incurvation. Both cornua measure 3.0–4.0 cm long. The uterus does not present an intercornual ligament, which distinguishes it from that in domestic ruminants. Each uterine cornu harbours four caruncles aligned in two rows.

**Figure 2:**
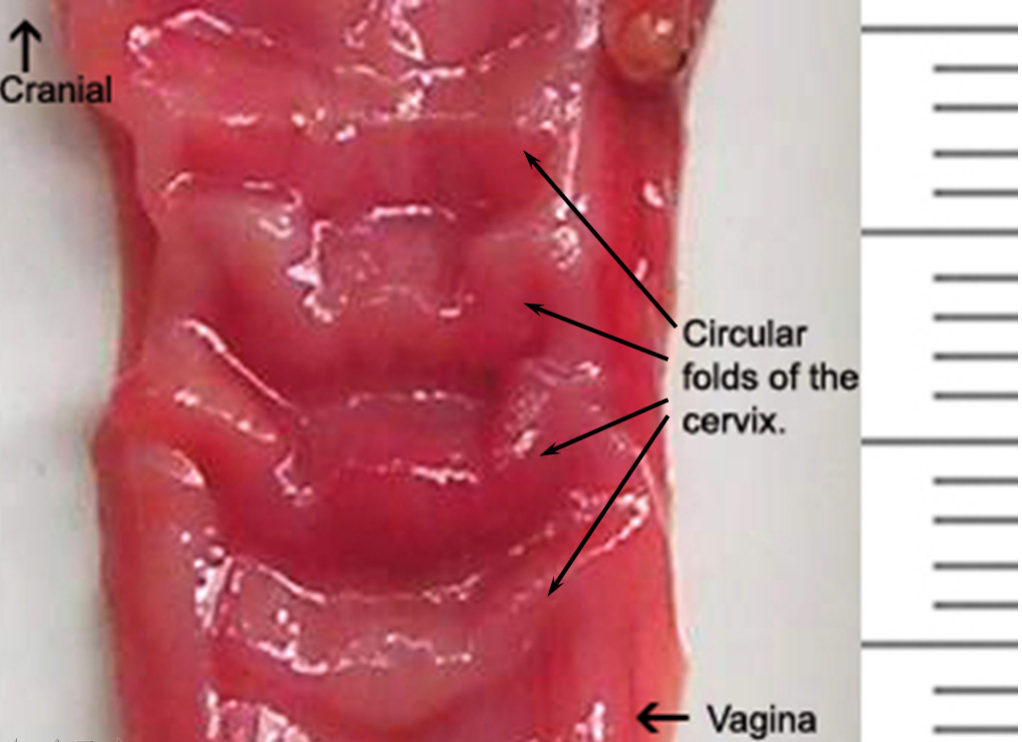
Circular folds projecting from the cervix into the cervical canal.

Distally, the cornua join together along a length of 1.0 cm, and look like a long corpus uteri. There are no caruncles in the corpus. The length of the corpus uteri is approximately 4.5 cm and its lumen is continuous cranially with the lumina of the cornua uteri through two discrete openings. The cervix is a firm tube that can readily be distinguished by palpation of the isolated reproductive tract from both the softer corpus uteri cranially and the vagina caudally. The cervix is 4.0 cm long, and 1.0 cm in diameter. Four circular folds project into the cervical canal (Fig. 2). These folds vary in height and length, and are annulus-shaped. The most cranial fold is not as well developed as the other three.

No vaginal part of the cervix can be distinguished. The vagina and vestibule are similar to those in small domestic ruminants. The vaginal mucosa forms longitudinal folds. The vulvar labia and the clitoris are not prominent. Overall, the anatomy of the female reproductive tract differs in some important respects, as reduced uterine length, uterine tubes flexuosity and small ovaries, from that of domestic ruminants.

### Puberty, oestrous cycle and gestational length

Few data are available on age of puberty in female pampas deer. Hinds born at the ECFA gave birth for the first time at approximately 21 months. If we consider that gestational length is probably no longer than 7–7.5 months (see below), the first fertile oestrus of hinds born at the ECFA is therefore at the age of no more than 14 months. We also observed a delay in age at first parturition in females originally captured from the wild. This was probably a consequence of the stress of capture, weaning and transportation [26].

There are no direct data regarding oestrous cycle length. González-Sierra [27] has suggested that cycle length is approximately 21 days, but presents no information on how those data were obtained (i.e. number of animals studied, method of recording oestrous behaviour, etc).

The gestational period in the species has been reported to be "a little longer than 7 months" [28]. Other authors agree that gestational length is between 7 and 7.5 months [20]. However, such data have been calculated in wild populations after roughly recording the period during which more sexual activity is observed and the period of the year in which more newborns are observed. As individual data are not available, this should be considered a general trend.

In other cervids, gestational length varies widely according to environmental conditions [29]. We expect that gestational length in the pampas deer may be even shorter than 7 months, according to some previous observations at the ECFA [19]. However, we should consider that at the ECFA, animals receive enough food throughout the year, including the gestational period. Therefore, if gestational length varies in pampas deer in relation to food availability, perhaps the gestational length at the ECFA is shorter than in wild populations.

### Birth and the early postpartum period

Some deer, such as the white-tailed deer, can have twins or even triplets [30-32]. However, like red deer and some other species [33,34], pampas deer generally deliver just one fawn [27,35]. A fawn's body weight at birth is approximately 2 kg [19]. At least under semi-captive conditions the body weight at birth is similar between male and female fawns, and is not influenced by season, mother's parity, or time since the previous parturition [19]. Delivery of twins was seen once [19], but both fawns died immediately after birth. At Emas National Park, in Goiás, Brazil (18°S/52°W), Redford [35] observed one female with two similar-sized young.

The sex ratio at birth is close to 1:1 in pampas deer [19]. However, some authors observed male:female ratios from 1:1.1 to 1:2 in adults [36], which suggests different mortality rates from birth to adulthood. Clutton-Brock et al. [37] observed a relationship between female parity, as well as date of parturition [37], and sex ratio in offspring of red deer. Gaillard et al. [38] did not find any difference in birth date at the year between the sexes in roe deer, suggesting that the sex ratio in pampas deer is more similar to that in roe deer than in red deer. An alternative explanation is that Clutton-Brock et al. [37] studied red deer in wild populations, while our study was done in a semi-captive population in which there are no changes in food availability throughout the year. Therefore, environmental pressures may have a stronger effect on the reproduction of these wild populations, differently affecting males and females.

One or two days before calving, pregnant females become restless and may start walking constantly, which has been reported in other deer species [30]. We observed that this period is also characterized by the elevation of the tail and losses of amniotic and birth fluids. Parturition occurs approximately 45–90 minutes after the initial leaking of birth fluids. During this time the hind is commonly seen repeatedly lying down and standing up, isolated from the rest of the group (unpublished data). As in other "hider" species of deer, the parturient female looks for and chooses a hidden and isolated place to lie down and deliver. Foetal forelegs appear visible in the doe's genitals approximately 1 hour before birth, at which time the hind is still walking and lying down repeatedly. As the intensity of the contractions increases, the hind lies down to deliver and the calf is born after approximately 15 minutes [Moore and Müller-Schwartze, unpublished draft available from the authors, [39]].

We have observed females standing up before the complete delivery of the fawn. Immediately after parturition, the mother intensively and continuously licks the fawn, eating all the amnion that surround it [27], similarly to what has been described in other deer species [30]. The placenta is released 1 hour after the fawn is born, and is also eaten [27,39]. The hind pulls the placenta out of her reproductive tract with her teeth and starts eating it before it is completely expelled (unpublished observations). During parturition it is not unusual to see other females approaching the parturient hind to smell the amniotic fluid and membranes extruding from her genitals, indicating the attraction that these fluids have for other members of the herd [39].

### Maternal behaviour and early fawn development

Almost immediately after birth, the fawn moves its tongue out of its mouth and makes head movements similar to those performed while searching for the udder. The fawn starts suckling 30–60 minutes after birth and also stands up at approximately that time. However, as in many other deer species, standing is not necessary for starting to suckle as the mother usually lies next to the fawn, and can start nursing it in this position. Pampas deer fawns can move from the site of birth, before standing up, by kicking the ground with their legs. When they do the mother follows the newborn and keeps licking it. The mother stays close to or lies at the birth site during the first hours after parturition and maintains frequent contact with the newborn.

While nursing, the mother licks the fawn, in particular its perianal region, stimulating urination and defaecation. During this initial time, mother and fawn learn to recognize each other. Although the establishment of a bond and recognition in this species has not been studied in detail, olfactory cues and vocalizations are probably critical, as in other deer species [29,40]. After having nursed the fawn for a short time the mother can leave the area of calving. Deer fawns hide and spend most of their first day of life separated from their mothers, who nurse them for just a few minutes during the first few postnatal days. Then, depending on the deer species [32], either the calf follows the mother (e.g. in reindeer) or the mother visits the site where the calf is hidden two to three times a day (red and pampas deer). In pampas deer, the encounters between the mother and the fawn change significantly over time. At least in semi-captive animals, during the first 2 weeks postpartum, the mother visits the site where the fawn is hidden, and calls it to attract it. Older fawns increase the frequency of searches for their mother in order to start nursing. Even so, fawns will continue emitting high-pitched vocalizations to call the mother to the place where they are hidden.

The mother hind calls the fawn by repeated brief, low-pitched vocalizations. In response to these calls, the fawn stands up without vocalizing during the first days postpartum. Part of the process of reciprocal recognition by the mother and fawn includes smelling each other, and making snout contact. The fawn emits high-pitched vocalizations when at risk, increasing the arousal of the herd. In such cases only the mother will approach in response to the alert call. Although reciprocal recognition between mother and offspring, as in other ungulates (e.g. sheep), is well developed in most deer species [40], allosuckling, and allomothering or adoption are not uncommon in fallow and red deer [41,42]. Therefore, fawns that do not receive enough milk from their mothers will usually look for milk from other lactating females. We do not know how common allosuckling or adoption is in pampas deer, but at least on one occasion we observed one fawn suckling for a short time from a different mother, simultaneously with her own fawn.

**Figure 3:**
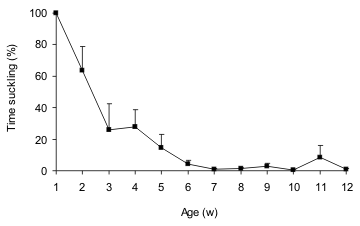
**Percentage of time spent suckling in relation to total feeding time (grazing, eating ration, leaves, or water abbreviating).** Behaviour of 18 fawns was recorded during 2 hours per week (1 hour in the morning and 1 in the afternoon) at the ECFA (unpublished data).

Deer fawns usually stay hidden but by 2–3 weeks of age they can run and play. At 4 weeks of age, they already graze but stay close to their mother most of the time. Weaning occurs at approximately 4 months of age [31,32]. However, in semi-captive individuals we observed that the time spent suckling in relation to total feeding time decreases at 3–6 weeks of age (Fig. 3). Beyond this period of maternal dependence, the mother and fawn keep a close relationship, showing affiliative behaviour such as mutual licking for several years. When a second fawn is born this relationship reduces in intensity and the mother starts rejecting the older fawn.

## Male reproduction

### Anatomy of the male reproductive tract

**Figure 4:**
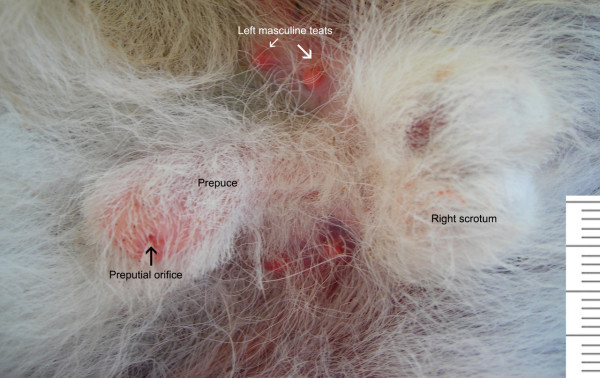
Prepuce and scrotum in male pampas deer (*Ozotoceros bezoarticus*).

**Figure 5:**
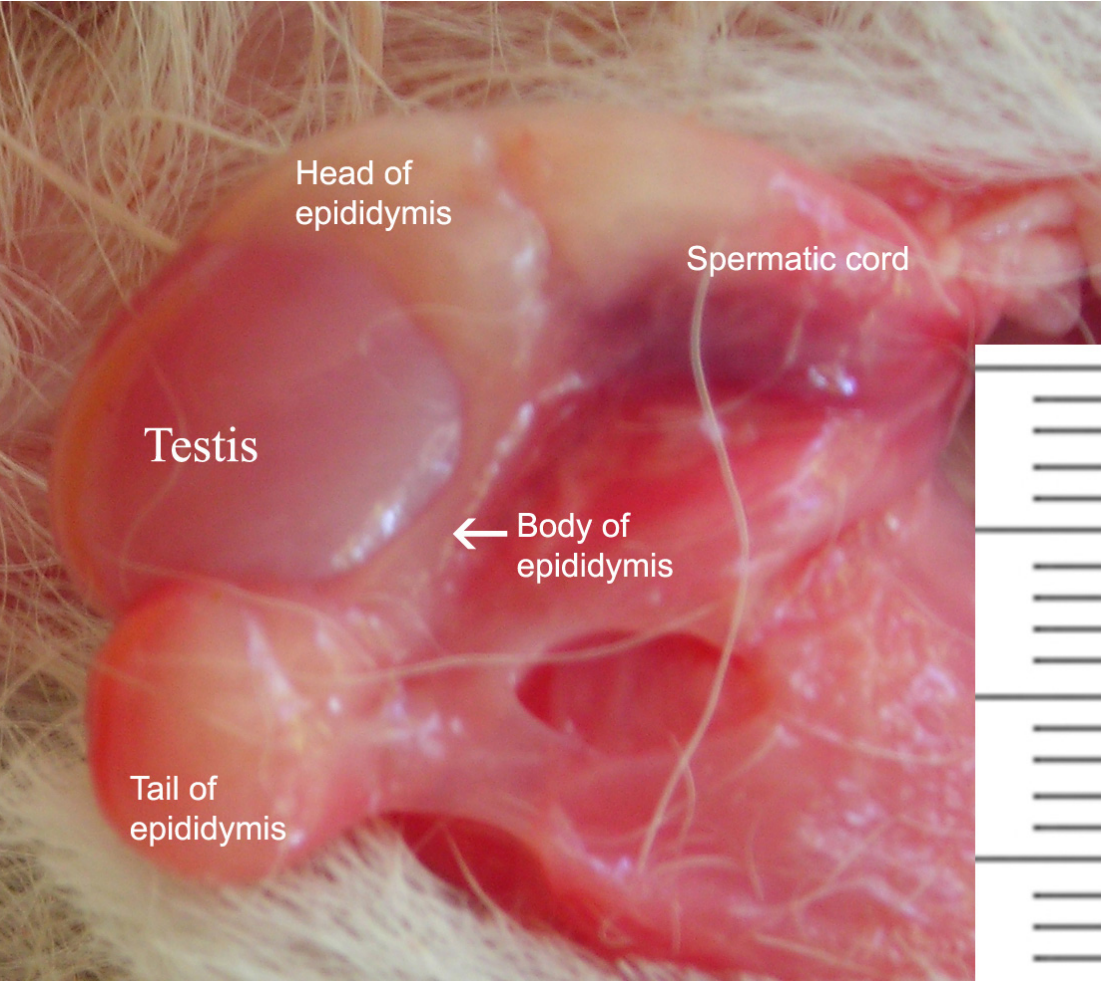
Testis, epididymis and spermatic cord of a prepubertal pampas deer (*Ozotoceros bezoarticus*).

The following description corresponds to four males (*O. bezoarticus arerunguaensis*) which died from natural causes at the ECFA in Uruguay. Their general anatomy is similar to that described for other deer species [43]. The prepuce and scrotum (Fig. 4) are covered with fine hairs. As in the female, two pairs of nipples are located on either side of the prepuce. The prepuce is a single fold consisting of external and internal laminae. The penis has a radix, corpus and glans. Its radix attaches to the ischial arches by two rounded crura. The penis is fibro-elastic, with no sigmoid flexure. The urethra is surrounded by a thin corpus spongiosum composed of erectile tissue, almost to the terminal external orifice. The unpigmented scrotum is attached near the body in the inguinal region, covered by the thighs. The deferent ducts are united by a genital fold containing blood vessels. The testis, epididymis and spermatic cord in a prepubertal male are presented in Figure 5.

The following sex glands can be recognized: the ampullae of the deferent ducts, the vesicular glands, the prostate and the bulbo-urethral glands. However, a well-defined corpus of the prostate is not observed.

### Male sexual development

Very little is known regarding male reproductive physiology in pampas deer. Male sexual development in pampas deer is mainly known from studies of antler development. During their first months, male fawns grow small, single-spiked, 2–8 cm long antlers (observations made at the ECFA, 2007). According to Whitehead [44], this phenomenon is uncommon in cervids, being described for some few species such as roe deer (*Capreolus capreolus*) and reindeer (*Rangifer tarandus*), and having been noted in *O. bezoarticus *in captivity in Berlin Zoo [39]. Preliminary observations from the ECFA report that fawns that were born in spring are not sexually mature by the following autumn. However, these individuals do show agonistic behaviour towards other males when 1 year old, i.e. by late spring. Moreover, according to González-Sierra [27], 1-year-old males show an interest in females during their second summer of life. We have also observed display of male courtship behaviour in 5–6-month-old males. However, it is difficult to determine whether this corresponds to playing or sexual behaviour. Therefore, puberty probably occurs at approximately 1 year of age.

## Seasonal reproductive pattern

### Seasonal distribution of births

It is well known that environmental conditions may importantly influence the reproductive strategies of ungulates [45-48]. Deer show seasonal reproductive patterns, although reproductive strategies to synchronize parturition with best survival probability may differ among deer species, as happens with most ungulates. Parturition in temperate climate species (e.g. mule deer [49], Eld's deer [50], musk deer [51], Père David's deer [52], red deer [33] and roe deer [38]) occurs in spring-summer. Seasonal reproductive patterns of wild ruminants may be influenced by photoperiod [53], population density [54], short [55] or long-term [47] effects of climate, physical condition during the rutting period [46], plant phenology [56], or socio-sexual stimuli [57,58].

In pampas deer, fawning periods seem to vary according to subspecies and location (see Table 1 in Merino et al. [59]) although all reports show a peak in fawn births beginning in August-October (southern hemisphere spring equinox: late September). In general terms, it can be said that in pampas deer populations inhabiting subtropical to temperate locations (*O. bezoaorticus celer *in Buenos Aires Province, in Argentina, and *O. bezoarticus arerunguaensis *and *O. bezoarticus uruguayensis *in southeastern Uruguay), births can occur all year round, and the fawning peak falls either in spring (Uruguay) or in late spring to late summer/early autumn (Buenos Aires Province, Argentina), roughly coinciding with pasture abundance peaks. The population in San Luis, Argentina (66°00' 34° 22'S), at the southwestern limit of the pampas deer range, inhabit dry grasslands with an average rainfall of 450 mm, 80% of which falls during October-April, a shade maximum temperature of 40°C in the summer and a winter minimum of -15°C [60]. Interestingly, the San Luis population (*O. bezoarticus celer*) which occur nearly exactly at the same latitude as the population in southeastern Uruguay, but in a semi-arid, continental climate, have a shorter fawning peak in spring. Furthermore, the fawning period does not extend through the year, but extends from late winter to late summer/early autumn. On the other hand, *O. bezoarticus bezoarticus *inhabit Cerrado do Pantanal region in Brazil, under tropical conditions where food availability varies more markedly than in the abovementioned habitats, due to sharp differences between the rainy and dry season. Consequently, the fawning period does not extend through the year, but is limited to July-November, with a peak in August-September, which coincides with the beginning of the rainy season.

**Figure 6:**
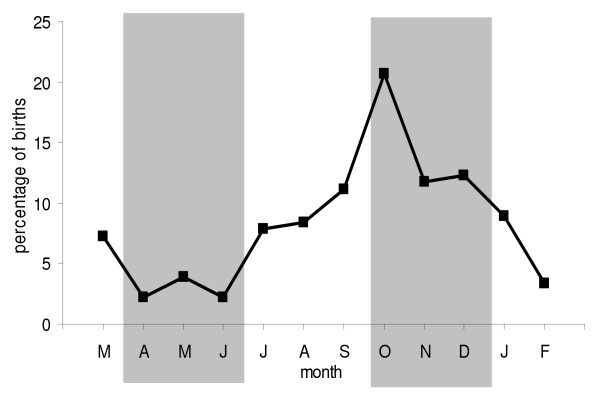
**Monthly distribution of birth percentages in a herd of semi-captive pampas deer (*Ozotoceros bezoarticus*) at the ECFA, Pan de Azúcar, in southern Uruguay, in a pool of 272 births recorded over 20 years.** Grey areas correspond to autumn and spring. Adapted from Ungerfeld et al. [19].

Redford [35] has suggested that pampas deer are similar to axis deer in not having a defined rutting season. However, most data suggest that although the seasonality is not as strong as in other deer species (e.g. white-tailed, roe or red deer), there are differences in the frequency of births observed throughout the year, suggesting an extended reproductive season and a high plasticity to respond to short-term signals. Moreover, food supply seems to be an important regulator of seasonality also in pampas deer. In captive pampas deer taken from Paraguay to Germany (Berlin Zoo, 52°30' N), Frädrich [39] reported no fixed rutting season, although males presented an annual antler cycle. However, how transportation and adaptation to a new environment with inverse seasons (due to the change of hemisphere) affected seasonality in that population remains unknown. We have analysed the distribution of 272 births that occurred during 20 years in a semi-captive population of pampas deer in Uruguay (34°3' S), where animals receive the same amount of food throughout the year [19]. Although births were observed every month, the distribution through the year suggests the existence of a seasonal reproductive pattern (Fig. 6). The seasonal pattern seems to be less rigid in semi-captive conditions than in wild populations from similar latitudes, suggesting that food availability has a direct influence on cyclic activity.

In our population at the ECFA we also observed that the parturition of primiparous females is more concentrated than parturitions of multiparous females [19]. Similar to many wild mammals [61-64], the age at first mating (primiparous females) is influenced by a body mass threshold, which is less limiting for multiparous females. Therefore, as is the case with roe deer [65], red deer [66], Alaskan moose [47] and Alaskan caribou [46], growth may be a major determinant of primiparity in pampas deer as females need to reach a threshold body mass to become pregnant. Since in the ECFA population, food is supplied in similar amounts throughout the year, it seems that photoperiod has a stronger effect on puberty attainment than on the cyclic activity of adult females. This difference in seasonal effects between pubertal and adult females is in agreement with the differences found in males between first antler cycle and the adult antler cycle.

Taking into consideration all this information, the pampas deer seems to be a seasonal breeder, with most parturitions occurring during spring, but with breeding activity possibly occurring throughout the year. There seems to be a photoperiod-induced seasonal cycle, which is strongly influenced by pasture availability.

### Seasonality in males

The male reproductive cycle of cervids in temperate latitudes is well known. These species are markedly seasonal, with important changes in antler cycle, neck musculature and testicular size and histology in the male [67,68]. Pampas deer males seem to be moderately seasonal. Given that at the ECFA, births have been recorded all year round (see above), it can be inferred that at least some males also breed all year round. However, a rutting season has been recognized in pampas deer from both Uruguay and Brazil. In Uruguay, the rutting season corresponds to February-April, and is defined as the period when male sparring and fighting is most frequently seen.

#### Antler cycle

As in other deer species, antlers are renewed every year. Undoubtedly, yearly antler renewal implies a high energy cost for males. Antler cycle is influenced by testicular androgen secretion [69]. In turn, this is influenced by photoperiod, melatonin and prolactin secretion [70,71]. Moreover, antler cycle is also influenced by nutrition, age, presence of parasites, and illness [72]. Antlers are cast from March to May at Emas National Park, with antlers in velvet starting to appear in April. Antler growth takes 30–45 days [[73]; González-Pensado & Villagrán, unpublished data], with maximal velvet presence during June and July and velvet shedding in August [74]. Jackson & Langguth [1] report that in San Borombón, Argentina, over 85% of males were in hard antler between January and July. Conversely, from August to November 70% of the males were in velvet. The breeding season coincides with hard antlers and males with antlers in velvet are likely to be less active [75].

In San Luis, Argentina, antlers are normally cast in August-September, towards the end of the southern winter. Antler regeneration starts immediately and by October-November all bucks are in velvet. The new antlers are generally full-sized and clean by December. In a recent study in the region, during February-April, which corresponds to the height of the rut, all 24 bucks studied had polished antlers. Rutting activity is concentrated in, but is not exclusive to, late summer and autumn [76]. Factors such as nutrition, age, presence of parasites, diseases and wounds may all influence antler cycle timing [77]. The San Luis data show a similar timing to that described by Jackson & Langguth [1] for the eastern groups of *O. bezoarticus celer*, with the major events in the cycle occurring about 1 month ahead of those reported for the region by Bianchini & Pérez [78].

In La Corona and Bahía de Samborombón, in Buenos Aires Province in Argentina, the majority of males are in hard antler from November-December through to July [79]. Antlers are cast around July and are cleaned of velvet by the end of the year. Bianchini & Pérez [78] concluded that in La Corona and Campos del Tuyú, shedding takes place between June and September, younger animals being the first to lose their antlers. During October, November and December, all deer are in velvet, which is stripped by late January/early February, with the antler cycle being 1 month later than reported by Jackson & Langguth [1]. Climatic conditions in Sierra de los Ajos, Rocha, Uruguay, are similar to those in Buenos Aires Province in Argentina [80]. All males there are in hard antler until July and antlers are shed in August. Antler growth begins quickly thereafter. Antlers are in velvet until November (with 50% of antlers in velvet, and 50% of antlers being hard during November). In December, all males are in hard antler.

**Figure 7:**
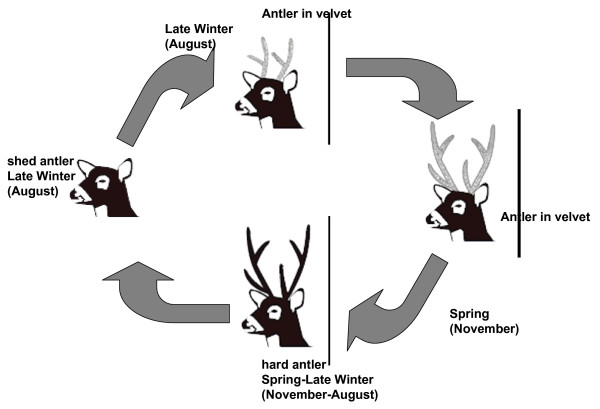
**Antler cycle in adult and yearling pampas deer males.** Adapted from Ungerfeld et al. [83].

Recent detailed data on 22 bucks and 72 antler cycles have been obtained in Uruguay at the ECFA [81]. The mean date of the first casting of antlers was 4 August (i.e. mid-winter). The brow and the trez tine were first observed 22.8 ± 0.6 and 45.9 ± 0.9 days after the first antler casting. Velvet shedding was observed 103.3 ± 2.1 days after the first antler casting. Both antler castings were observed later in first-antler cycle bucks than in adult bucks, although the interval between both castings was not different. The interval from the brow tine observation to the trez tine observation was reported to be shorter in first-antler cycle bucks than in adults. As in other deer species, the antler cycle was reported to be seasonal, but unlike most deer species, cycle to cycle antler growth persisted well into adulthood. Figure 7 summarizes the antler cycle through the year in adult and yearling males.

#### Seasonal changes in male reproduction

In Brazil at Emas National Park, *O. bezoarticus bezoarticus *displays a biannual cycle in testosterone faecal concentration, with a summer peak corresponding to the major rutting season and another peak observed in winter-spring, which is a period of antler mineralization and velvet shedding. Semen quality in *O. bezoarticus bezoarticus *in Brazil (at Pantanal Matogrossense and Emas National Park) was reported to be regular in February but of low quality in July and September [82]. However, one out of six animals studied, having antlers in velvet, produced good quality semen in July.

Reproductive behaviour has been correlated to testosterone faecal concentration in *O. bezoarticus bezoarticus *[74]. There are two peaks in reproductive activity, in December-January (summer) and in July-September (winter-spring). Anogenital and urine sniffing, flehmen, chasing, fighting and mounting are the prevailing behaviours of the first peak, while gland marking is the most frequently observed behaviour during the second peak. Conversely, mean group size is highest in September (4.2 animals/group), with the lowest means in October-February (mean range 1.7–2.7 animals/group) [74].

## Reproductive behaviour

### Courtship behaviour

**Figure 8:**
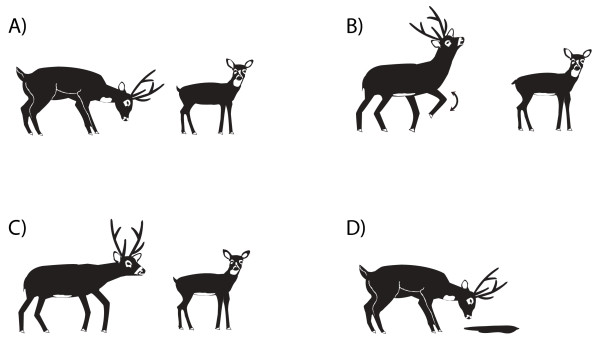
**Courtship behavioural events in pampas deer: A) low stretch; B) ostentation; C) anogenital sniffing; and D) smelling urine.** See detailed descriptions of each behaviour in Table 1.

The most complete description of courtship behaviour in pampas deer has been reported by Verdier [83] in free-ranging *O. bezoarticus uruguayensis *in Rocha, Uruguay. He described seven courtship behavioural units, which are defined in Table 1. We have also observed that after a low stretch (Fig. 8A), the male lies down near the female and may stay there for several hours, either as a kind of "guard" to determine receptiveness of the female or to keep other males away. Similar behaviours have been reported in mouflons (*Ovis aries*) [84].

**Table 1:** Courtship behavioural units observed in pampas deer (*Ozotoceros bezoarticus*).

Behavioural unit	Behaviour in the male
Low stretch	Walks toward the female with his head and neck stretched, the head held in a low position, a few centimetres above the ground, maintaining a distance of 2–10 m
Ostentation	Stands immobile in front of the female, sometimes walking a few steps toward her. The neck is held erect and the head horizontal, generally rotated to one side. The chin is held up high.
Anogenital sniffing	Pushes its face into the female's anogenital region.
Chivying	Marches towards the female, with the neck in a normal position.
Chivying and nodding	Similar to chivying, but sporadically moves the head and neck up and down, keeping them still for no longer than 1 second
Smelling urine	Smells and puts the nose in the female's urine
Flehmen	The head is raised, the external nares are drawn back, and the upper lip is curled back.

Likewise, we have observed ostentation (Fig. 8B), which is always performed < 3 m away from the female, and which also includes the alternate movements of the front legs. These are flexed slowly, kept flexed for a few seconds in the air and then extended briskly, thumping the soil violently. This movement is performed alternately with both front legs. Interestingly, Thomas et al. [85] define a similar behavioural unit, the "hard look", in the repertoire of agonistic behavioural units of the white-tailed deer.

Anogenital sniffing (Fig. 8C) in mouflons has also been described as "sniffing the rear" [84] while in domestic sheep it has been described as nudging [86]. Chivying is performed at a greater distance than the low stretch. Clutton-Brock et al. [33] describe this behaviour in red deer. We have recently confirmed Frädrich's observations [39] that males may vocalize simultaneously.

We also confirmed Frädrich's [39] observation that the male smells and puts his nose in the female's urine (smell urine, Fig. 8D). In some cases we saw males gently rubbing the soil where the female had just urinated. Furthermore, some males hastily approach the female when she adopts the urinating position and will wet their nose with the urine before it reaches the soil.

**Figure 9:**
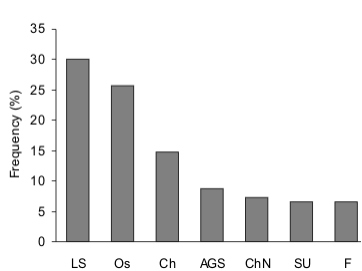
**Frequency of each courtship behavioural unit in pampas deer (*Ozotoceros bezoarticus*).** LS = low stretch; Os = ostentation; AGS = anogenital sniffing; Ch = chivying; ChN = chivying and nodding; SU = smelling urine; F = flehmen. See detailed descriptions of each behaviour in Table 1. Redrawn from Verdier [83].

Figure 9 shows the frequency at which each behaviour was observed by Verdier [83], the low stretch and ostentation being the most common behaviours. As has been observed in other ruminants [86], smelling urine and flehmen are the only behaviours that are highly associated.

Vos et al. [87] state that masturbation by deer has not been recorded. However, recently at the ECFA we observed one adult male masturbation, with strong pelvic movements during approximately 5 seconds while chivying. This occurred in January. The male was kept in a pen with cycling females. However, masturbation did not end in visible ejaculation.

### Mating

In our observations, the male walks toward the female with his head and neck stretched, with the head in a low position (low stretch), and begins to smell the female's anogenital region. Next, the male approaches laterally, beginning with tongue movements, similar to the ram's nudge [86]. The female displays proceptive behaviours, such as nibbling the male's ear, or walks towards the male, and lifts and moves her tail near the male's nose, inviting the male to discover her vulva. Finally the male mounts and mates her. We have also observed incomplete and lateral mounting of receptive females by males. This has also been reported in other deer species [33,34], mouflon [84] and sheep [86]. However, we observed this only once in one sexually inexperienced male. During mating, the male displays strong and quick movements during 3–4 seconds. We observed six to ten pelvic movements. After mating, the male has a short refractory period during which the female may walk a few metres away while the male stays immobilized. However, the female then lifts her tail and exposes the vulva lips contracting, repeatedly exposing her clitoris.

### Chemical communication and male reproduction

Social behaviour including reproduction is profoundly influenced by chemical signals in ruminants, including deer [for a review, see Gosling, [88]]. We have attempted to summarize the sources of chemical signals, and some of the most common behaviours used to transmit or receive them. The most common secreting pathways reported for deer are urine, faeces, saliva, and secretions from specialized cutaneous glands [88]. Scent marks are commonly placed in the environment, on their own bodies, or on the body of other individuals. Different strategies have been reported in different deer species.

The forms and possible functions of the cutaneous glands in *Ozotoceros bezoarticus *from Uruguay have been described by Langguth & Jackson [89]. The preorbital gland is situated in a depression of the external face, near the lacrimal bone, and extends forward from the anterior corner of the eye. The tarsal gland is located on the inner side of the tarsal joint, and the interdigital gland is located between the first phalanges of the two principal toes in the hindfoot. The forehead and the bases of the antlers are stiffer than the surrounding areas, and furthermore, the sites corresponding to the bucks' antler stumps or bases are marked in females by two white dots [89].

Both sexes have the above mentioned glands but only males display marking activity. An adult male in hard antler has an outer orifice of the preorbital gland with a diameter of 1.5 cm. The substances secreted by the cutaneous glands are left on a wide variety of substrates. Pampas deer males exhibit marking behaviour associated with each of the three cutaneous glands. Verdier [83] describes the marking behaviour of pampas deer both in the wild and in captivity (Table 2).

**Table 2:** Marking behavioural units displayed by pampas deer males (*Ozotoceros bezoarticus*).

Behavioural unit	Behaviour in the male
Frontal marking	Brisk swinging head motions, either lateral or vertical, rubbing both antlers, simultaneously or alternately. Frequently performed with the tail raised, showing the perineal region.
Rubbing	A beating and twisting of the brush and small limbs, initially in the stripping of the velvet and consequently, although perhaps incidentally, in the polishing of the antlers
Antler trashing	The chafing of the basal antler parts against the trunks of small trees
Pre-orbital marking	Stands with the neck stretched, pressing the gland against an object, with short and energetic up and down movements
Front leg marking	Performed with the neck slightly below the shoulder height, scratching the ground with the front hooves, frequently alternating the hooves

**Figure 10:**
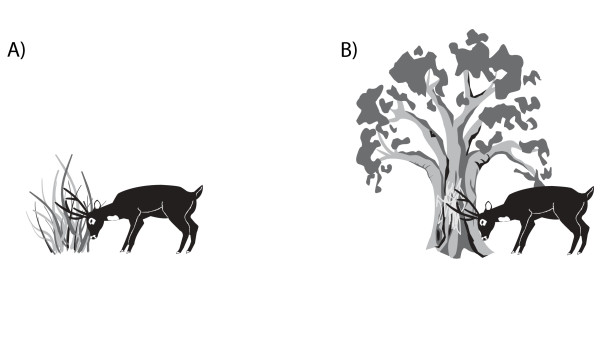
Frontal marking on A) tufts of grass; and B) tree branches.

Frontal marking is the most frequently observed marking in both adult and juvenile males. The places chosen to perform this behaviour are tree branches, bushes or grass tufts (Fig. 10). The movements are generally performed in series, every few seconds or every few steps. Frontal marking may be associated with marking with front legs [83].

Rubbing has also been described in mule deer [Lindsdale & Tomich, 1953, cited by de Vos et al. [87]], red deer [33] and pampas deer [76]. Altmann [90] states that the rubbing of antlers by elk is an erotic stimulus. Darling [91] notes that among European red deer, antler rubbing is an auto-erotic stimulus. Denniston [92] considers antler rubbing and masturbation a part of the sex drive response complex of moose.

Antler trashing, which has been observed only in adult males in hard antler [76], results in very visible barkless zones in trees, approximately 40 cm long [39]. Mock fights with vegetation are often associated with antler thrashing. White-tailed deer bucks expend much energy during the rut in fighting any object viewed as an obstacle or opponent [93]. Linsdale & Tomich [94] propose that these fights are practice runs for real contests, but they also state that the rubbing of antlers "may have become so formalized that it is often engaged in for its own sake as the actual contests may be".

Preorbital marking is performed with the preorbital gland. The sequence of movements is repeated immediately with the contralateral gland. Free-ranging pampas deer generally mark tree branches [83], but our observations at the ECFA have been that males normally perform this behaviour less frequently than other types of marking behaviour. Pampas deer at the ECFA have also been seen to mark cement columns.

Front leg marking was seen by us in only one adult male with velvet antlers. Both semi-captive (our own observations) and free-ranging pampas deer [83] perform this behaviour with the tail up and curved over the back. The male then frequently urinates the recently marked ground, resulting in a patch of ground without grass (50 cm), which is, however, not easily visible.

Males kept in a pen with only females have also been observed challenging males separated from their pen by a 1 m wide corridor by performing frontal marking on bushes and small branches, and frontal marking on the wire fence right in front of the place where another male would be standing. Similar behaviour has been reported for reindeer [Espmark, 1964, referred to in de Vos et al., [87]].

### Male agonistic behaviour

Pampas deer have an established social hierarchy, maintained by aggressive-submissive behaviours [76]. Agonistic interactions, such as scent marking, increase in number during the pre-rut period. In confined male groups, individuals maintain distances of > 5 m from each other, except during feeding time, a period during which an important number of agonistic interactions are observed. This limit may correspond to the "critical distance" of approximately 3 m described by Altman [95] in elk (*Cervus canadensis*) and moose (*Alces alces*).

At the ECFA, we observed similar behavioural events to those described in the wild to dominate other individuals (Table 3).

**Table 3:** Agonistic behaviours displayed by pampas deer males (*Ozotoceros bezoarticus*)

Behavioural unit	Description of the interaction
Glare	The dominant male stands with the neck held erect and looks steadily at the other individual, with the ears occasionally dropped flat against the neck.
Antler present threat	The antler points in the direction of the other individual, with the ears back and dropped. May be part of sparring.
Nose-touching contact	With the neck erect, the noses of two males make contact.
Shaking head	Dominant males raise their neck and shake their head.
Head low threat and chase	The head and the neck are lowered, with the ears held back.
Strike and barge	Using its forefoot the male strikes another individual on the shoulder or the croup.
Sparring and fighting	These behaviours are not easily distinguishable.

**Figure 11:**
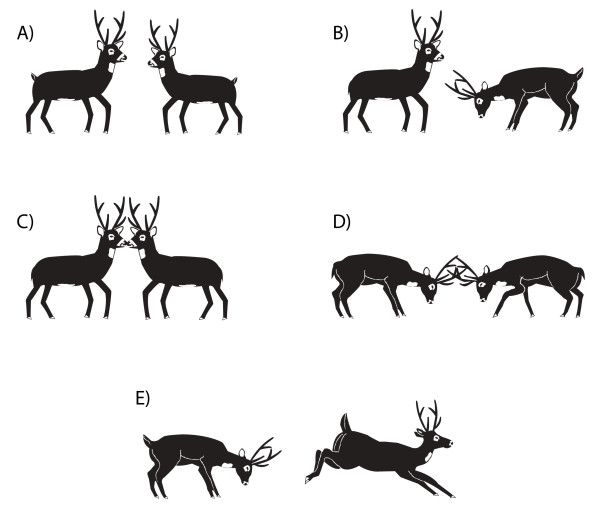
**Male agonistic behaviours in male pampas deer(*Ozotoceros bezoarticus*): A) glare; B) the antler presentthreat; C) nose-touching; D) locking of antlers; E) pursuing asubordinate individual. **See detailed descriptions of each behaviour in Table 3.

Glare (Fig. 11A) leads to submission without physical interaction. Consequently, it is commonly observed in established groups of males, although it has also been observed towards females and young males. Antler present threat is presented in Fig. 11B. Nose-touching contact (Fig. 11C) is commonly observed in most social interactions. It has been suggested that it is related to individual identification [76,89].

While the shaking of the head is similar to what has been observed in *Odocoileus hemionus crooki *[96], the head low threat and chase is similar to the "hard look" reported in white-tailed deer (*Odocoileus virginalis texanus*) [85]. The last is followed on some occasions by short chases. As in the wild [76] it is the most common agonistic event we have observed in semi-captivity.

We observed that when one male strikes and barges, the submissive individual quickly escapes. In red deer, two males confronting each other have been observed to stroke with their forefoot while standing on their hind legs. Although we have not observed this behaviour in adult males, we have in a young hand-reared male (5 months) toward other unfamiliar young males. Jackson [76] also saw it in one hand-reared animal toward its owner.

Sparring and fighting have often been observed between young males, and not so frequently between adult males. While in young males these behaviours have been observed in both animals in hard antler and animals with velvet antlers, in adult males they are more common in individuals in hard antler. Jackson [76] also observed that young males commonly initiate sparring and contests against mature males. According to Verdier [83], fights can be divided up into three stages: onset, course, and conclusion. During the onset the aggressor approaches a rival, generally glaring at it and nose-touching. Jackson [76] observed that males might then gently touch each other's antler tips, before locking their antlers, to push back and forth, twisting and turning for up to 3 minutes (Fig. 11D). During this period males look steadily at each other, and push steadily. After the 3 minutes, it is common to stop pushing, and glance or nouse-touch again before beginning another period of locking antlers, pushing back and forth, twisting and turning again. Sometimes fights end when males unlock their antlers and one of them or both walk away from each other. Bianchini & Perez [78] and Jackson [76] report that submissive individuals are not pursued, although we have occasionally observed persecutions (Fig. 11E). Commonly, individuals do not engage again, but walk slowly 1 m apart for 10–30 m, presenting their antlers and sparring [76], similarly to reports in *Dama dama *[97] and *Cervus elaphus *[33]. We have also observed walking similar to that described by Jackson [76] between males that were not in direct contact, but separated by fencing. Sometimes several males challenge the same or nearby bucks. As reported for fallow deer [97], it is frequently difficult to determine who is the winner of the fight [[83], and our own observations].

Commonly submissive individuals just walk away, with the ears back and up, or expose the neck [76]. Frädrich [39] observed the head lowered and slow walking away from the aggressor. Possibly the lack of physical contact in most of behavioural units as well as the small number of combats observed between adult males contribute to "a lower frequency of fight" and "decrease [the] risk of serious injury", as reported by McElliot et al. [cited by Bartos, [97]] in *Dama dama*.

## Reproductive technologies: semen collection and cryopreservation

### Semen collection

South American deer populations are dwindling. Reproductive biotechnology techniques such as artificial insemination (AI), in vitro fertilization (IVF) and embryo transfer have been used effectively in animal conservation [98,99] and specifically in other wild deer [100]. Semen from pampas deer has been collected by electro-ejaculation, artificial vagina and vaginal collection.

Electro-ejaculation is undoubtedly the method of choice in untamed animals and it is the most frequently used semen collection technique in deer [82]. Jaczewski & Jasiorowski [101] used electro-ejaculation for semen collection in *Cervus elaphus *(using a voltage of 4.5–31.5 V, amperage 0.2–0.7 A). Semen collection from free-ranging *O. bezoarticus leucogaster *males was performed successfully by Duarte et al. [102]. Duarte & Garcia [103] successfully used the equipment habitually used for bulls with an electrode modified to fit pampas deer anatomy. This technique was used for *Ozotoceros*, *Mazama *and *Blastocerus*. Animals should always be sedated (1–2 mg/kg xylazine and 5 mg/kg ketamine) either intramuscularly or intravenously. The males were stimulated with 250–750 mA, resting every 3 seconds until ten stimuli had been performed. After a 1–2-minute rest the procedure was repeated. No more than three stimulation sessions were performed per collection [103]. The semen volume obtained is very small (0.1–0.7 mL) but the concentration is high (mean 1,500 × 10^6 ^spermatozoa (spz)/mL; maximum 3,000 × 10^6 ^spz/mL). No more than one weekly collection should be performed.

In pampas deer we obtained semen from anaesthetized males with electro-ejaculation using electric discharges during 3 seconds and resting for 2 seconds. Series of ten stimulating periods were repeated, beginning with 1 v and ending with 6 v. Males ejaculated 0.1–0.45 mL during 5 and 6-v series. In some occasions, ejaculates were obtained without erection.

Pampas deer semen has also been obtained with an artificial sheep vagina [103]. More frequent collections can be performed using this method. However, this technique can only be used in tame, trained animals. Stuffed animals can be used, with female urine poured onto the dorsal region. In very tame animals with strong imprinting, i.e. animals that have been raised by humans since birth, collection can be performed on the operator's knee [103]. A doll fitted with an artificial vagina is used for shy animals. The doll is left inside the deer's enclosure and the operator just waits for the animal to copulate [103] as described for red deer by Krziwinski & Bobek [104]. Soto et al. [105] collected semen on the knee of an operator from one *O. bezoarticus celer *male born in captivity. They used "Danish-type" artificial vaginas at a temperature of 42°C. Ejaculated volumes using this method are very small (0.1–0.25 mL).

In Santa Fe, Argentina, semen in anaesthetized males was obtained by electro-ejaculation, using increasing pulse series from 2 V, until ejaculation. Overall, semen was obtained seven times from five different males (three times during mid-winter, twice in early autumn, once during early summer and once during mid-spring). One or two fractions were obtained in all animals. Volumes varied from 0.1 mL to 0.6 mL. The percentage of motile spermatozoa ranged from 20% to 70%, and concentration varied from 15 to 1,235 million/mL. Total spermatozoa/ejaculate varied from 34 to 130 millions (A.J. Sestelo, N.L. Jácome, M.A. Rivolta, V. Astore, personal communication).

### Semen evaluation

Semen should be evaluated after collection. Semen volume is measured in a scaled centrifuge tube with a conical end. An aliquot should be taken for formol-saline fixation, in order to measure sperm concentration at a dilution of 1:200. Several diluents have been used for cervids. Haigh et al. [106] tried three diluents made up of citrate and egg yolk (20%), skimmed milk (2%) and promide-D (vegetable protein) to freeze and thaw *Cervus elaphus *semen. Diluents were tested with or without adding 0.2% sodium ethylenediaminetetra-acetic acid (EDTA). Glycerol as cryoprotectant and antibiotics to impair bacterial growth were added to all diluents. The EDTA-added diluent proved to be best, both because of a lower loss of sperm motility and because of fewer acrosomal defects after thawing. Monfort et al. [107] working with Eld's deer (*Cervus eldi thamin*) frozen-thawed spermatozoa using a diluent made up of 1.2 g hydroxymethyl methyl-2-amino ethane sulphonic acid (TES-n-tris), 1.2 g hydroxymethylaminomethane (Tris), 1.6 g glucose, 1.6 g fructose, 1.5 mL amino-sodium-lauryl sulphate, 20 mL egg yolk, 0.04 g penicillin G and 0.1 g streptomycin in 100 mL diluent. The decrement in semen motility after freezing-thawing was only 20–30%. Duarte & Garcia [108] used Tris-citric acid buffer (4.54% Tris, 2.6% citric acid, 0.75% glucose) with 2.25% egg yolk and 6% glycerol in several Brazilian deer species, including *O. bezoarticus bezoarticus*. However, better results were achieved later by the same authors by adding 10% egg yolk. According to Duarte & Garcia [102], glycerol should preferably be added to the diluted semen during the final dilution, to minimize sperm damage before freezing due to the cryoprotectant. Prior to semen freezing, diluted semen is chilled to 4°C by placing the collection tube in a vessel containing warm water (35°C) and storing it at 4°C for 4 hours. Semen is then stored in 0.5 mL paillettes, each containing 50 × 10^6 ^spermatozoa. Chilled semen is placed over liquid N_2 _vapour (2–3 cm over N_2 _level) for 10 minutes before immersion into N_2_.

After copulation of a hind in heat, the doe is sedated and semen is collected with a Pasteur pipette. To minimize semen loss, an artificial mucosa should be introduced into the hind's vagina [101].

## Conclusions and future directions of research

The pampas deer has received less attention that it should, considering the limited distribution of the species. In particular, important aspects of the reproduction of this species are unknown. In this review we have summarized mainly anatomical and behavioural information on the species. Some of this information has already been published and studied systematically while some is preliminary evidence or describes single observations that require more research. However, one aspect that is mostly unexplored is the physiology of reproduction in pampas deer. Our research group aims to investigate all aspects of pampas deer reproduction so that this knowledge and the available modern reproductive techniques can be used to improve the situation of the species. Management and the use of appropriate techniques have been shown to be effective in improving reproductive rate in other ruminants. In this case, a strategy of conservation of pampas deer should include this type of intervention. Therefore, there is significant need to continue doing research in this species. Basic information such as the age at which pampas deer reach puberty, first oestrous cycle, and the gestational length have not been systematically determined. The availability of this type of information is critical to enable us to manage and intervene to improve reproductive rates in captive animals and the condition of the species for conservation in wild populations.

## List of abbreviations

CITES: Convention on International Trade in Endangered Species of Wild Fauna and Flora. ECFA: Estación de Cría de Fauna Autóctona; Native Fauna Breeding Station. UICN: International Union for Conservation of Nature and Natural Resources.

## Competing interests

The authors declare that they have no competing interests.

## Authors' contributions

RU began and coordinated the research performed at the ECFA, was SG-P's and MV's advisor, drafted the manuscript, and co-ordinated the writing and editing of the ms. SG-P wrote the courtship and mating behaviour sections, helped directly in writing the sections on agonistic behaviour, marking, and birthing and the early postpartum period, as well as the maternal behaviour and early fawn development sections. SG-P also collected important unpublished data included in the manuscript. AB wrote the sections dealing with seasonal reproductive patterns and reproductive technologies, i.e. semen collection and cryopreservation. AB also reviewed and helped with most sections, and collected unpublished data. MV wrote the section on male agonistic behaviour, and the chemical communication and male reproduction sections, and helped writing the courtship and mating behaviour, birth and early postpartum, and maternal behaviour and early fawn development sections, as well as collecting important unpublished data included in the manuscript. DO wrote the basic text on birthing and the early postpartum period, and on maternal behaviour and early fawn development. DO also helped to draft, and reviewed, the manuscript. WP collected the original data related to anatomical sections, and helped with the editing of the manuscript.
